# Supplementary material for: Tropinone synthesis via an atypical polyketide synthase and P450-mediated cyclization
Source: Nat Commun. 2018 Dec 11;9:5281. doi: 10.1038/s41467-018-07671-3 (PMC6290073; doi:10.1038/s41467-018-07671-3)
Supplement: Supplementary file 1 — Supplementary Information [file 41467_2018_7671_MOESM1_ESM.pdf]

Supplementary Information

Tropinone synthesis via an atypical polyketide synthase and P450-mediated cyclization

Bedewitz et al.,

Supplementary Figures

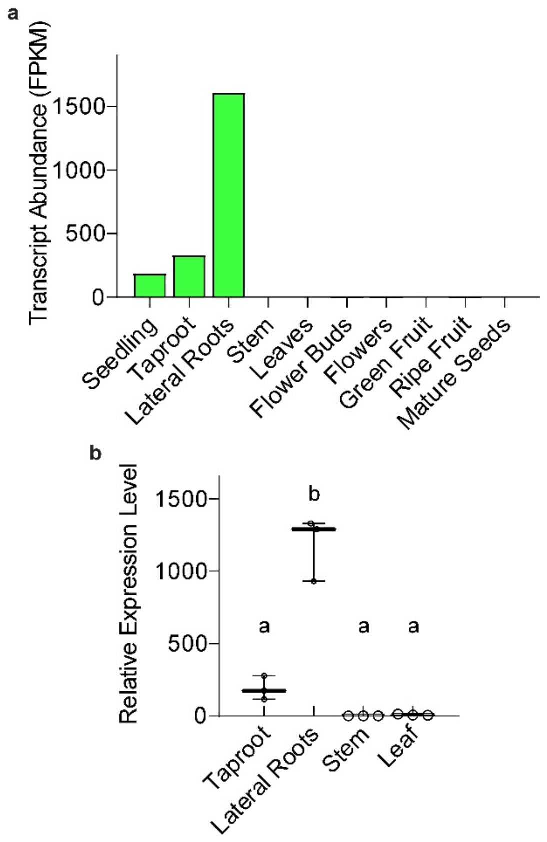

Supplementary Figure 1. Expression of a root-preferentially expressed type III polyketide synthase in *Atropa belladonna*. **a** Transcript abundance of aba_locus_1322 (*AbPYKS*) was determined by RNA-seq analysis using a previously described dataset [1] and is presented as Fragments per Kilobase of Transcript per Million Mapped Reads (FPKM). **b** Relative expression level of aba_locus_1322 (*AbPYKS*) in *A. belladonna* as determined by qRT-PCR. Data are presented as three biological and three technical replicates relative to the expression level detected in stem. Different letters indicate significant differences ($p \leq 0.05$) in expression level between tissue types, determined by a Brown-Forsythe and Welch One-Way ANOVA as described in the Statistical Analysis section of the Methods. For each box plot, the line represents the median value and the whiskers extend to the minimum and maximum data points. Source data are provided as a Source Data file.

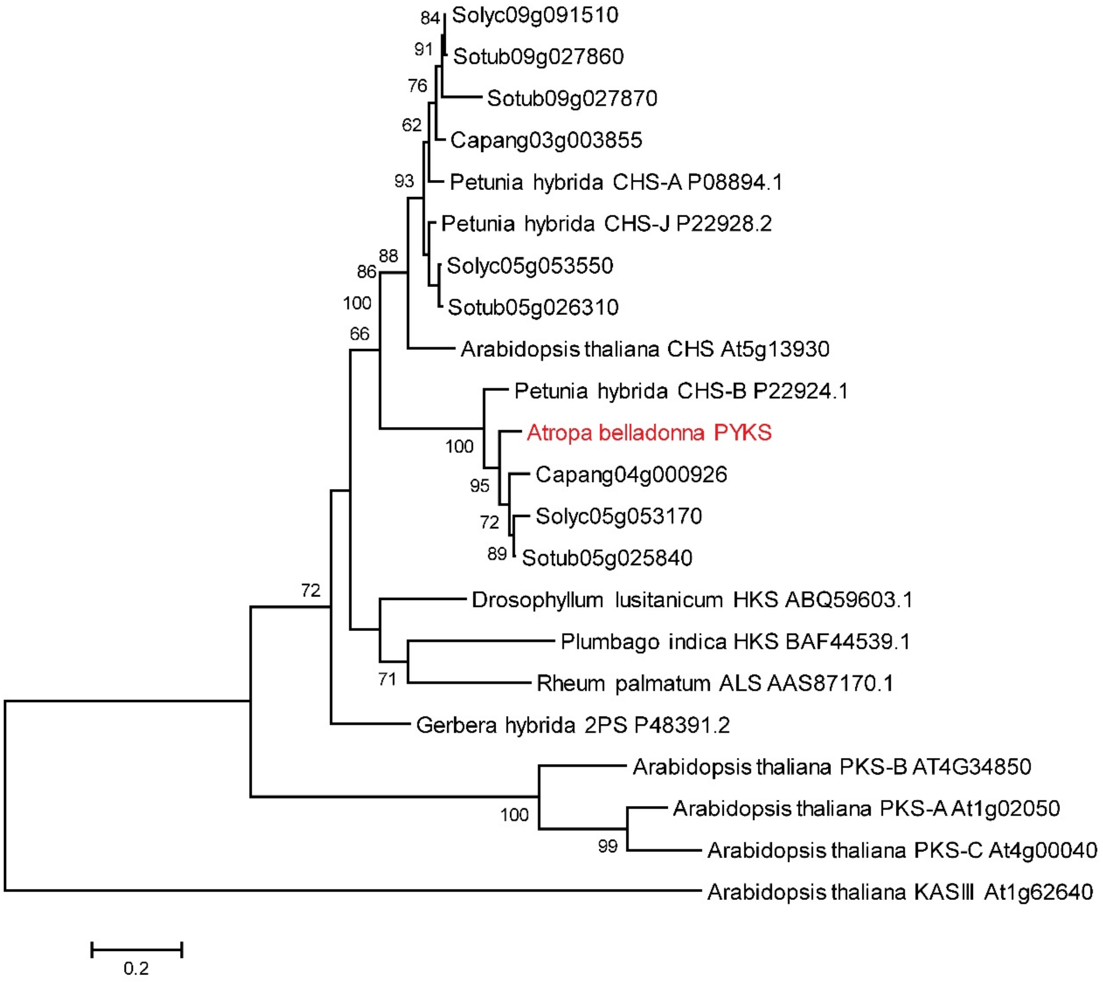

Supplementary Figure 2. Phylogenetic relationship of select plant polyketide synthases (PKSs). An unrooted maximum likelihood phylogenetic tree obtained using MEGA version 5 using a combination of functionally characterized and predicted PKS amino acid sequences. Bootstrap values greater than 50 derived from 2000 replicates are shown on the nodes of the tree. *PYKS* from *A. belladonna* is shown in red.

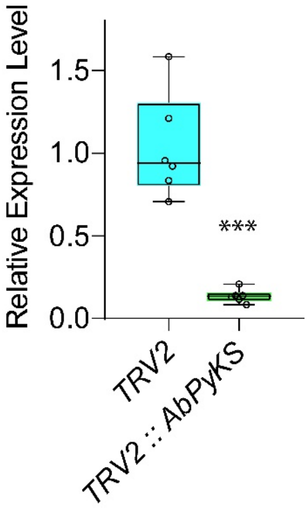

Supplementary Figure 3. Silencing efficiency in *Ab1322* VIGS lines. Relative expression level of aba_locus_1322 (*AbPYKS*) in *TRV2* empty vector and *AbPYKS* VIGS lines. Data are presented as six biological and three technical replicates with the expression level in *TRV2* empty vector control lines set to 1. The six plants selected for gene expression analysis represent individuals with tropinone levels closest to the median values for the given genotypes presented in (Fig. 1b). Asterisks denote significant differences (*** = p < 0.001) as determined by Student's *t* test. For each box plot, the lower and upper bounds of the box indicate the first and third quartiles, the line indicates the median value, and the whiskers extend to the minimum and maximum data points. Source data are provided as a Source Data file.

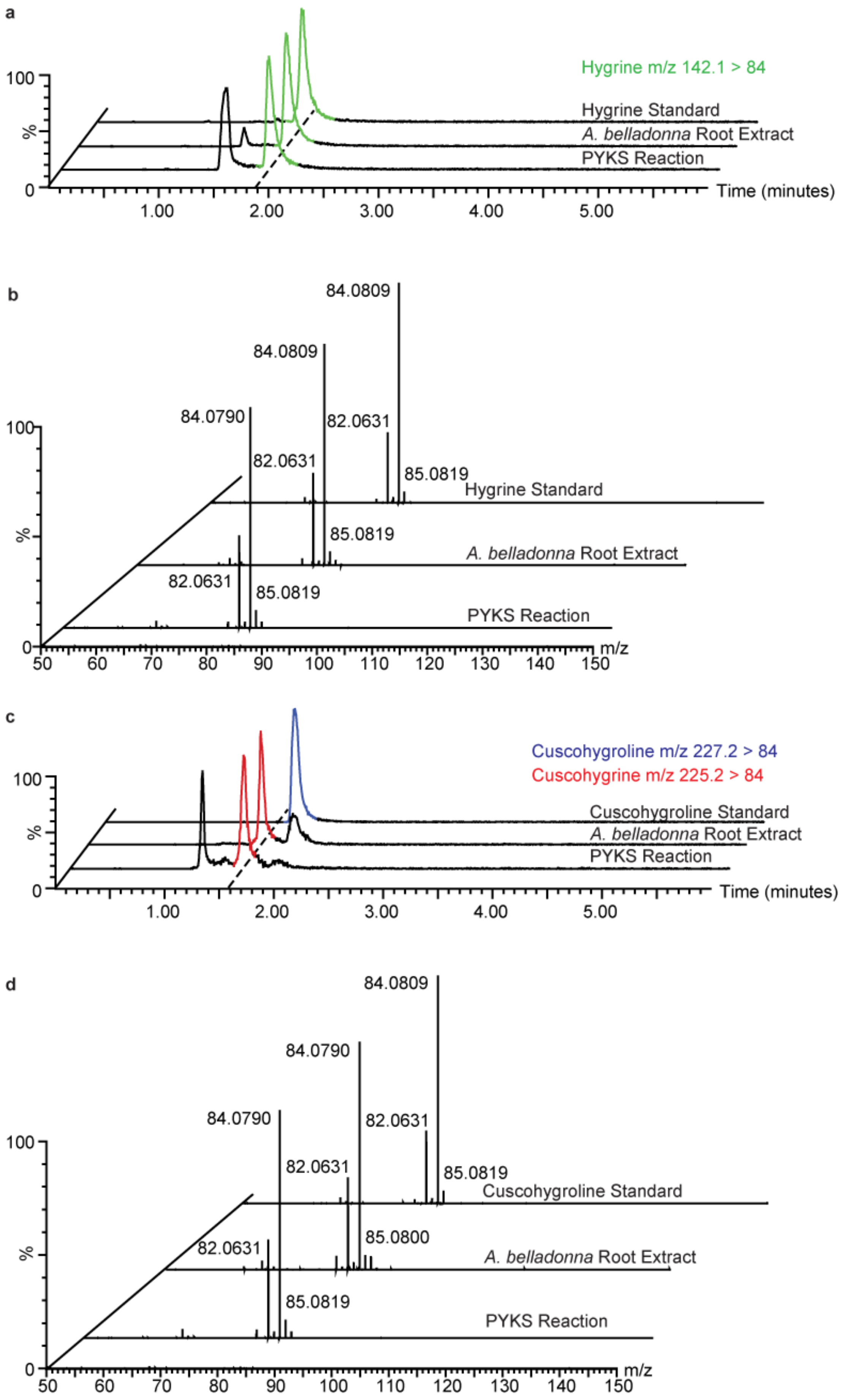
a
Hygrine m/z 142.1 > 84
Hygrine Standard
A. belladonna Root Extract
PYKS Reaction
100
%
0
1.00
2.00
3.00
4.00
5.00
Time (minutes)
b
84.0809
84.0809
84.0790
82.0631
82.0631
85.0819
Hygrine Standard
85.0819
A. belladonna Root Extract
82.0631
85.0819
PYKS Reaction
m/z
50
60
70
80
90
100
110
120
130
140
150
c
Cuscohygroline m/z 227.2 > 84
Cuscohygrine m/z 225.2 > 84
Cuscohygroline Standard
A. belladonna Root Extract
PYKS Reaction
Time (minutes)
d
84.0809
84.0790
84.0790
82.0631
82.0631
85.0819
Cuscohygroline Standard
85.0800
82.0631
A. belladonna Root Extract
85.0819
PYKS Reaction
m/z

Supplementary Figure 4. AbPYKS catalysis yields hygrine and cuscohygrine. a Extracted ion LC/MS/MS MRM chromatograms in positive-ion mode (*m/z* 142.1 > 84) for the hygrine $[M+H]^+$ ion from a hygrine standard, a root extract of a 5-week old *A. belladonna* plant, and a representative AbPYKS enzyme reaction. Hygrine is shown in green. **b** Product ion MS/MS spectra for *m/z* 142 ($[M+H]^+$) of hygrine in a hygrine standard, a root extract of a 5-week old *A. belladonna* plant, and a representative AbPYKS enzyme reaction using data-independent SONAR MS/MS acquisition as described in Methods **c** Extracted ion LC/MS/MS MRM chromatograms in positive-ion mode for the cuscohygroline (*m/z* 227.2 > 84) ion or the cuscohygrine (*m/z* 225.2 > 84) ion from an authentic cuscohygroline standard, a root extract of a 5-week old *A. belladonna* plant, and a representative AbPYKS enzyme reaction. Cuscohygroline is shown in blue, and cuscohygrine is shown in red. **d** Product ion MS/MS spectra for *m/z* 225 of cuscohygroline or cuscohygrine in a cuscohygroline standard, a root extract of a 5-week old *A. belladonna* plant, and a representative AbPYKS enzyme reaction under SONAR as described in Methods.

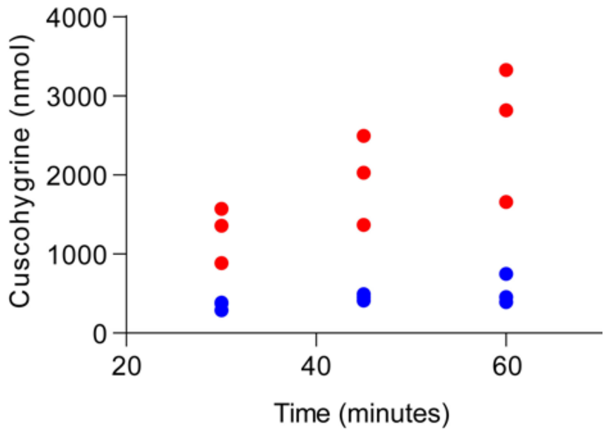

Supplementary Figure 5. *In vitro* generation of cuscohygrine. Nanomoles of cuscohygrine produced over time under two reaction conditions. Blue circles indicate reactions performed using a 1:1 ratio of *N*-methyl-Δ^1-pyrrolinium to 4-(1-methyl-2-pyrrolidinyl)-3-oxobutanoic acid. Red circles indicate reactions performed using a 48:1 ratio of *N*-methyl-Δ^1-pyrrolinium to 4-(1-methyl-2-pyrrolidinyl)-3-oxobutanoic acid, representative of apparent relative metabolite pool sizes in *AbPYKS* VIGS lines. Data are presented as n = 3 independent reactions sampled over time, and all data points are displayed. Source data are provided as a Source Data file.

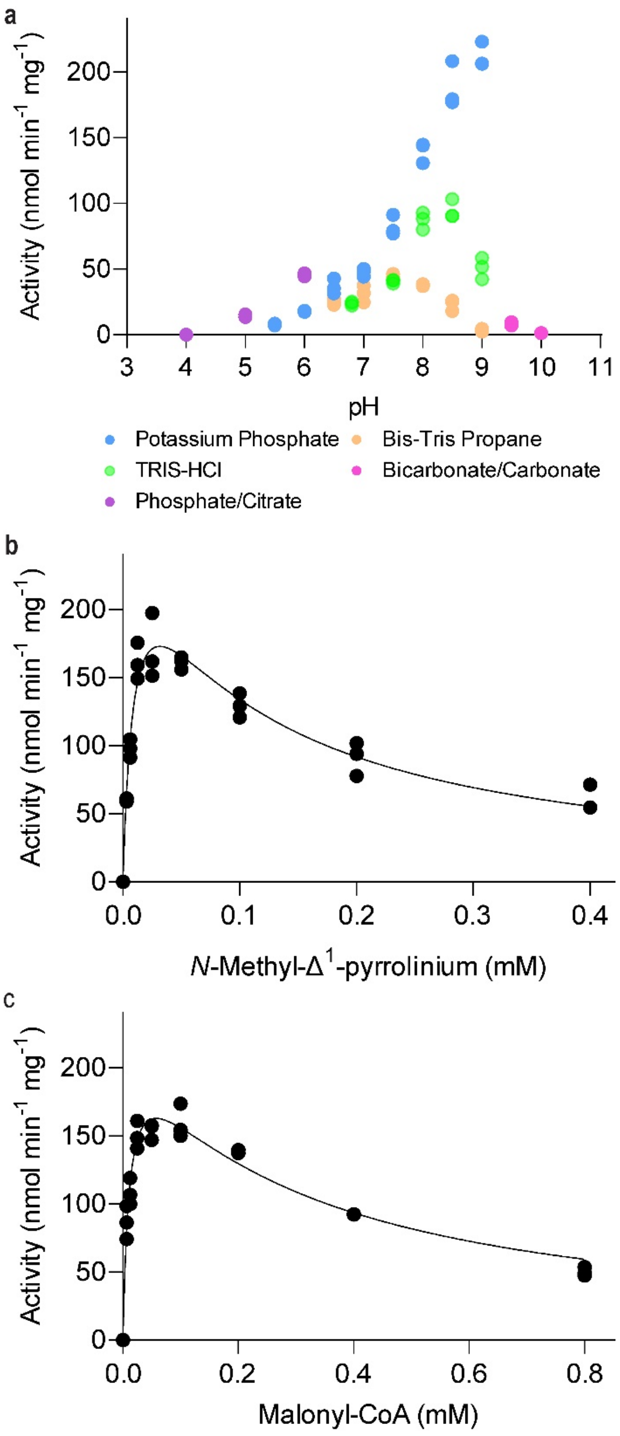

Supplementary Figure 6. pH optimum and enzyme kinetics for AbPYKS. a Determination of the pH optimum of AbPYKS. The different buffers used to span the pH range tested are indicated by blue, green, purple, sand, or magenta circles. **b** Determination of Michaelis-Menten enzyme kinetic parameters of AbPYKS for *N*-methyl-Δ^1-pyrrolinium using 0.1 mM malonyl-CoA as the co-substrate. Individual data points are displayed as black circles **c** Determination of Michaelis-Menten enzyme kinetic parameters of AbPYKS for malonyl-CoA using 0.05 mM *N*-methyl-Δ^1-pyrrolinium as the co-substrate. Individual data points are displayed as black circles Data are presented as n = 3 technical replicates for each pH condition or substrate concentration and all data points are displayed. Source data are provided as a Source Data file.

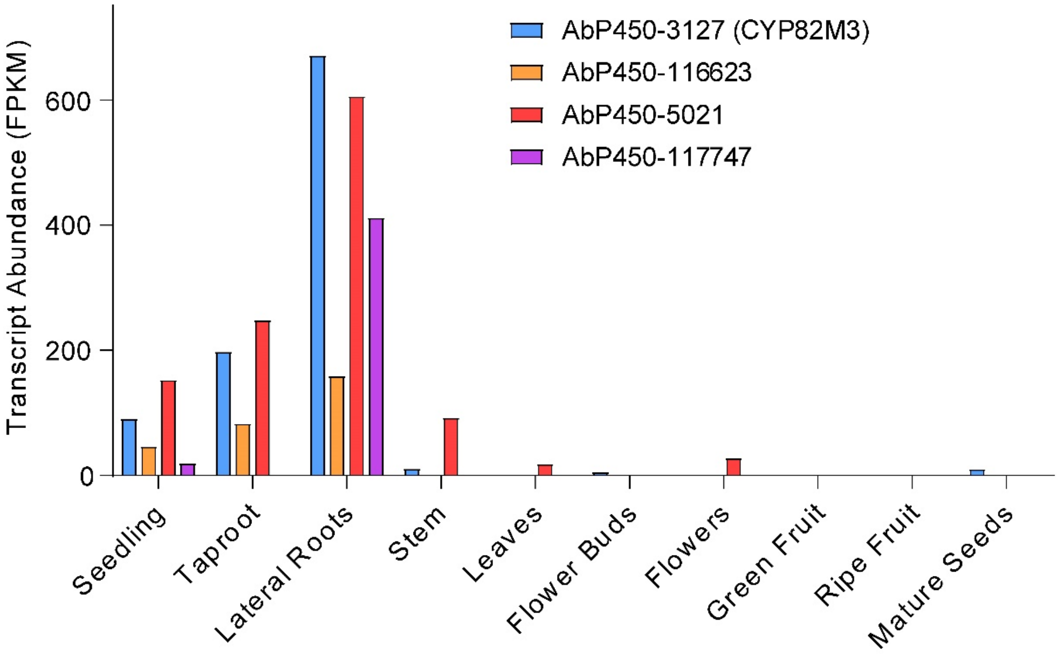

Supplementary Figure 7. Transcript abundance of root-preferentially expressed cytochrome P450s in *Atropa belladonna*. Transcript abundance of cytochrome P450 transcripts with a 5-fold or greater enriched expression level in lateral roots of *A. belladonna* compared to non-root tissues. Transcript abundance was determined by RNA-seq analysis using a previously described dataset [1] and is presented as Fragments per Kilobase of Transcript per Million Mapped Reads (FPKM).

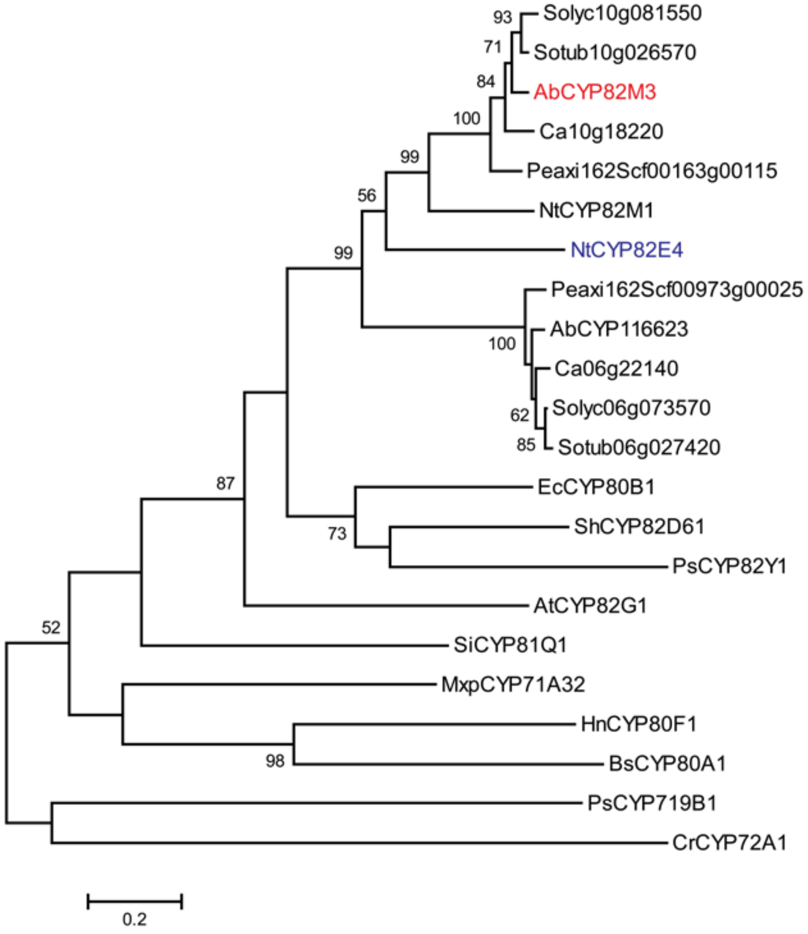

Supplementary Figure 8. Phylogenetic relationship of select plant cytochrome P450s (CYPs). An unrooted maximum likelihood phylogenetic tree obtained using MEGA version 5 using a combination of functionally characterized and predicted CYP amino acid sequences, with a preference for sequences catalyzing alkaloid related reactions or unusual ring closures. Bootstrap values greater than 50 derived from 2000 replicates are shown on the nodes of the tree. *AbCYP82M3* from *A. belladonna* are shown in red. Nicotine demethylase (NtCYP82E4) is shown in blue.

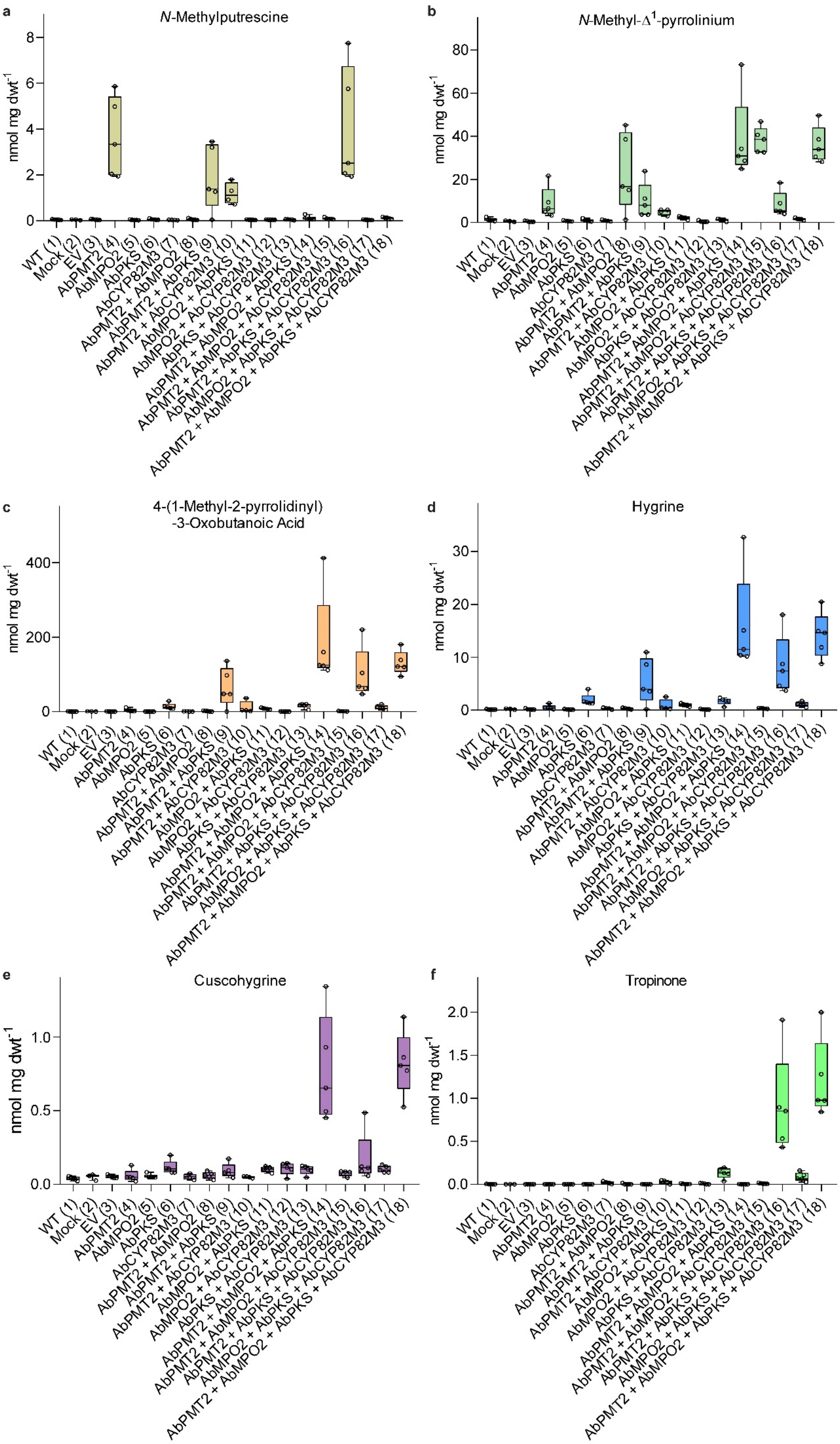

a
N-Methylputrescine
b
N-Methyl-Δ1-pyrrolinium
c
4-(1-Methyl-2-pyrrolidinyl)
-3-Oxobutanoic Acid
d
Hygrine
e
Cuscohygrine
f
Tropinone
nmol mg dwt-1
WT (1)
Mock (2)
EV (3)
AbPMT2 (4)
AbMPO2 (5)
AbPKS (6)
AbCYP82M3 (7)
AbPMT2 + AbMPO2 (8)
AbPMT2 + AbPKS (9)
AbPMT2 + AbCYP82M3 (10)
AbMPO2 + AbPKS (11)
AbMPO2 + AbCYP82M3 (12)
AbPKS + AbCYP82M3 (13)
AbPMT2 + AbMPO2 + AbPKS (14)
AbPMT2 + AbMPO2 + AbCYP82M3 (15)
AbPMT2 + AbPKS + AbCYP82M3 (16)
AbMPO2 + AbPKS + AbCYP82M3 (17)
AbPMT2 + AbMPO2 + AbPKS + AbCYP82M3 (18)

Supplementary Figure 9. The abundance of tropane precursors and pyrrolidine alkaloids in *Nicotiana benthamiana* leaves expressing AbCYP3127 (AbCYP82M3). Leaves of *N. benthamiana* were infiltrated with combinations of *Agrobacterium tumefaciens* strains individually transformed with the indicated constructs. Wild type (WT) uninfiltrated leaves, leaves infiltrated with infiltration solution without *A. tumefaciens* (Mock), and leaves infiltrated with the empty pEAQ vector (EV) were included as negative controls. Each panel **a** – **f** corresponds to a distinct metabolite. Data are presented as n = 5 biological replicates, except for (2) where n = 3, and (7) and (10) where n = 4. For each box plot, the lower and upper bounds of the box indicate the first and third quartiles, the line indicates the median value, and the whiskers extend to the minimum and maximum data points. Source data are provided as a Source Data file.

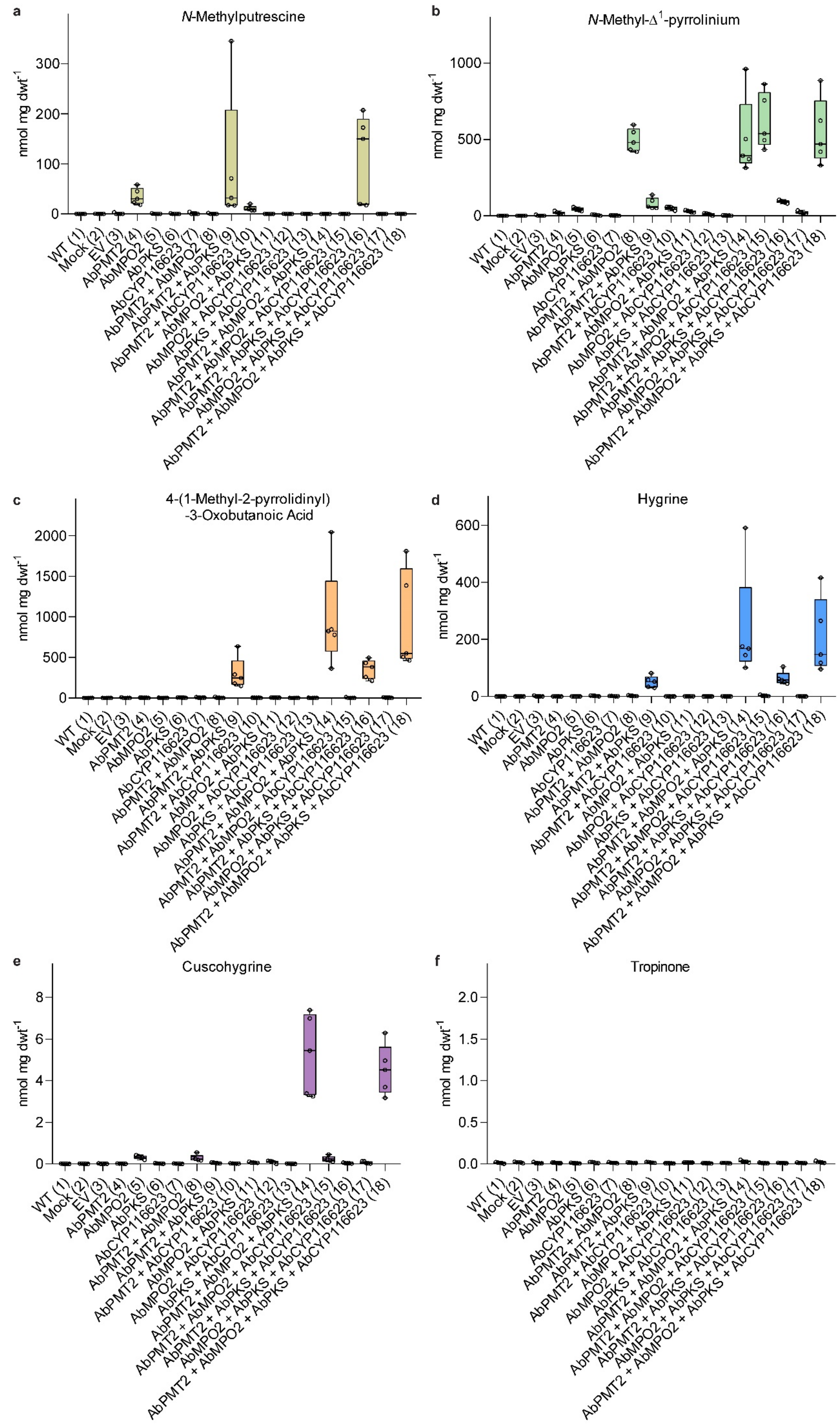
a
N-Methylputrescine
nmol mg dwt^{-1}
0
100
200
300
b
N-Methyl-Δ^1-pyrrolinium
0
500
1000
c
4-(1-Methyl-2-pyrrolidinyl)
-3-Oxobutanoic Acid
0
500
1000
1500
2000
d
Hygrine
0
200
400
600
e
Cuscohygrine
0
2
4
6
8
f
Tropinone
0.0
0.5
1.0
1.5
2.0
WT (1)
Mock (2)
EV (3)
AbPMT2 (4)
AbMPO2 (5)
AbPKS (6)
AbCYP116623 (7)
AbPMT2 + AbMPO2 (8)
AbPMT2 + AbPKS (9)
AbPMT2 + AbCYP116623 (10)
AbMPO2 + AbPKS (11)
AbMPO2 + AbCYP116623 (12)
AbPKS + AbCYP116623 (13)
AbPMT2 + AbMPO2 + AbPKS (14)
AbPMT2 + AbMPO2 + AbCYP116623 (15)
AbPMT2 + AbPKS + AbCYP116623 (16)
AbMPO2 + AbPKS + AbCYP116623 (17)
AbPMT2 + AbMPO2 + AbPKS + AbCYP116623 (18)

Supplementary Figure 10. The abundance of tropane precursors and pyrrolidine alkaloids in *Nicotiana benthamiana* leaves expressing AbCYP116623. Leaves of *N. benthamiana* were infiltrated with combinations of *Agrobacterium tumefaciens* strains individually transformed with the indicated constructs. Wild type (WT) uninfiltrated leaves, leaves infiltrated with infiltration solution without *A. tumefaciens* (Mock), and leaves infiltrated with the empty pEAQ vector (EV) were included as negative controls. Each panel **a** – **f** corresponds to a distinct metabolite. Data are presented as n = 5 biological replicates. For each box plot, the lower and upper bounds of the box indicate the first and third quartiles, the line indicates the median value, and the whiskers extend to the minimum and maximum data points. Source data are provided as a Source Data file.

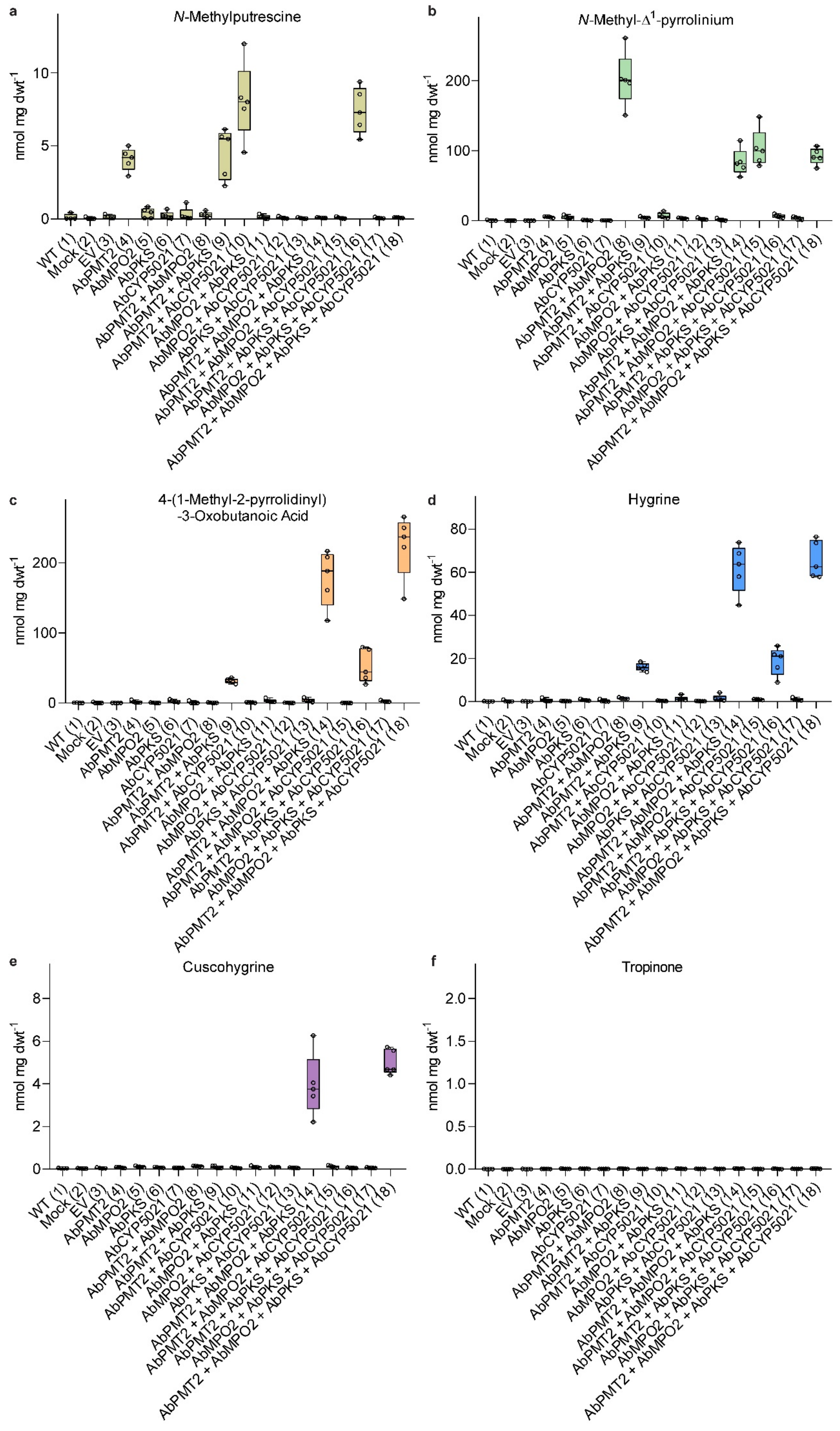

a
N-Methylputrescine
nmol mg dwt-1
0
5
10
b
N-Methyl-Δ1-pyrrolinium
nmol mg dwt-1
0
100
200
c
4-(1-Methyl-2-pyrrolidinyl)
-3-Oxobutanoic Acid
nmol mg dwt-1
0
100
200
d
Hygrine
nmol mg dwt-1
0
20
40
60
80
e
Cuscohygrine
nmol mg dwt-1
0
2
4
6
8
f
Tropinone
nmol mg dwt-1
0.0
0.5
1.0
1.5
2.0
WT (1)
Mock (2)
EV (3)
AbPMT2 (4)
AbMPO2 (5)
AbPKS (6)
AbCYP5021 (7)
AbPMT2 + AbMPO2 (8)
AbPMT2 + AbPKS (9)
AbPMT2 + AbCYP5021 (10)
AbMPO2 + AbPKS (11)
AbMPO2 + AbCYP5021 (12)
AbPKS + AbCYP5021 (13)
AbPMT2 + AbMPO2 + AbPKS (14)
AbPMT2 + AbMPO2 + AbCYP5021 (15)
AbPMT2 + AbPKS + AbCYP5021 (16)
AbMPO2 + AbPKS + AbCYP5021 (17)
AbPMT2 + AbMPO2 + AbPKS + AbCYP5021 (18)

Supplementary Figure 11. The abundance of tropane precursors and pyrrolidine alkaloids in *Nicotiana benthamiana* leaves expressing AbCYP5021. Leaves of *N. benthamiana* were infiltrated with combinations of *Agrobacterium tumefaciens* strains individually transformed with the indicated constructs. Wild type (WT) uninfiltrated leaves, leaves infiltrated with infiltration solution without *A. tumefaciens* (Mock), and leaves infiltrated with the empty pEAQ vector (EV) were included as negative controls. Each panel **a** – **f** corresponds to a distinct metabolite. Data are presented as *n* = 5 biological replicates, except (1) and (3), where *n* = 4. For each box plot, the lower and upper bounds of the box indicate the first and third quartiles, the line indicates the median value, and the whiskers extend to the minimum and maximum data points. Source data are provided as a Source Data file.

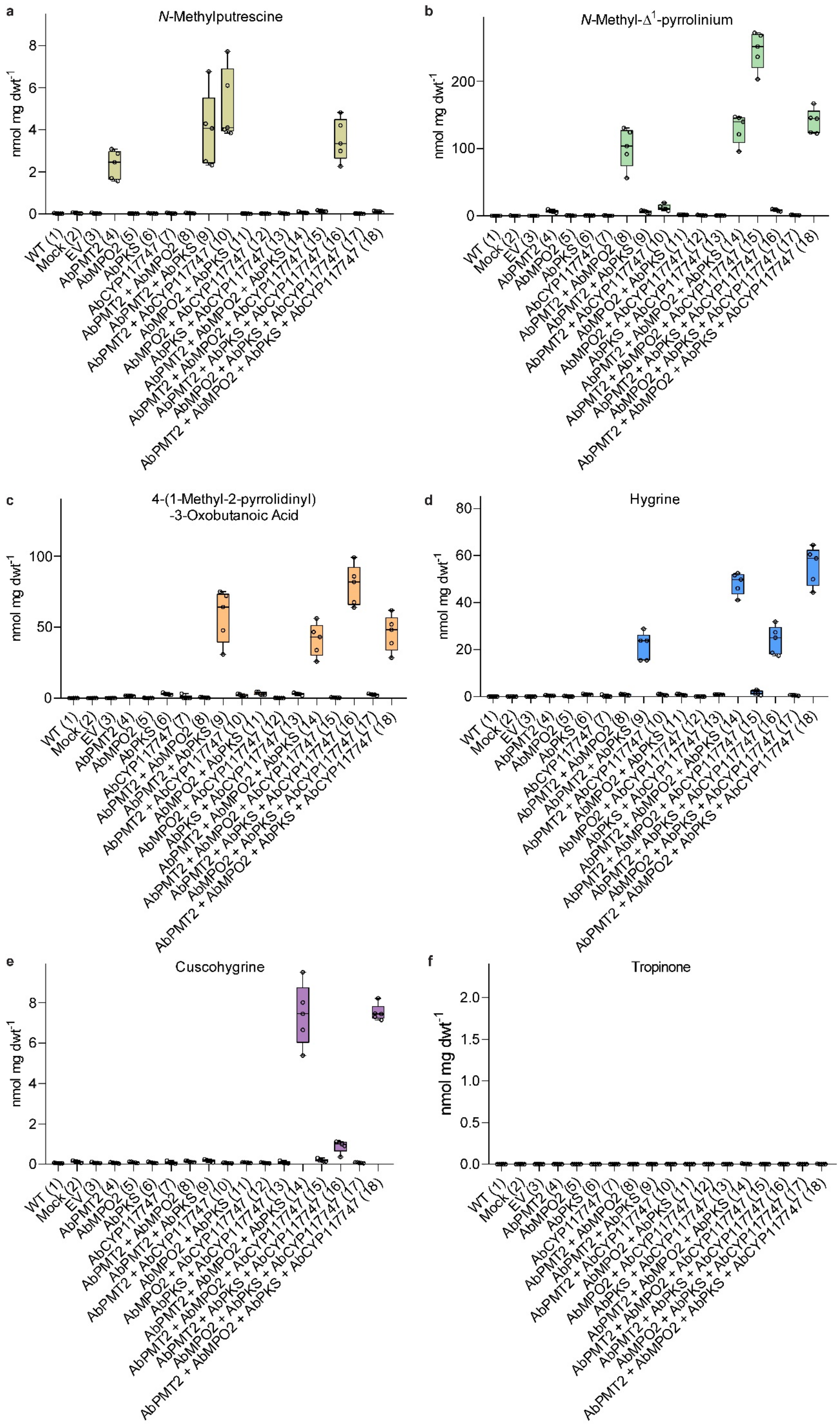
a
N-Methylputrescine
nmol mg dwt-1
b
N-Methyl-Δ1-pyrrolinium
c
4-(1-Methyl-2-pyrrolidinyl)
-3-Oxobutanoic Acid
d
Hygrine
e
Cuscohygrine
f
Tropinone
WT (1)
Mock (2)
EV (3)
AbPMT2 (4)
AbMPO2 (5)
AbPKS (6)
AbCYP117747 (7)
AbPMT2 + AbMPO2 (8)
AbPMT2 + AbPKS (9)
AbPMT2 + AbCYP117747 (10)
AbMPO2 + AbPKS (11)
AbMPO2 + AbCYP117747 (12)
AbPKS + AbCYP117747 (13)
AbPMT2 + AbMPO2 + AbPKS (14)
AbPMT2 + AbMPO2 + AbCYP117747 (15)
AbPMT2 + AbPKS + AbCYP117747 (16)
AbMPO2 + AbPKS + AbCYP117747 (17)
AbPMT2 + AbMPO2 + AbPKS + AbCYP117747 (18)

Supplementary Figure 12. The abundance of tropane precursors and pyrrolidine alkaloids in *Nicotiana benthamiana* leaves expressing AbCYP117747. Leaves of *N. benthamiana* were infiltrated with combinations of *Agrobacterium tumefaciens* strains individually transformed with the indicated constructs. Wild type (WT) uninfiltrated leaves, leaves infiltrated with infiltration solution without *A. tumefaciens* (Mock), and leaves infiltrated with the empty pEAQ vector (EV) were included as negative controls. Each panel **a** – **f** corresponds to a distinct metabolite. Data are presented as n = 5 biological replicates. For each box plot, the lower and upper bounds of the box indicate the first and third quartiles, the line indicates the median value, and the whiskers extend to the minimum and maximum data points. Source data are provided as a Source Data file.

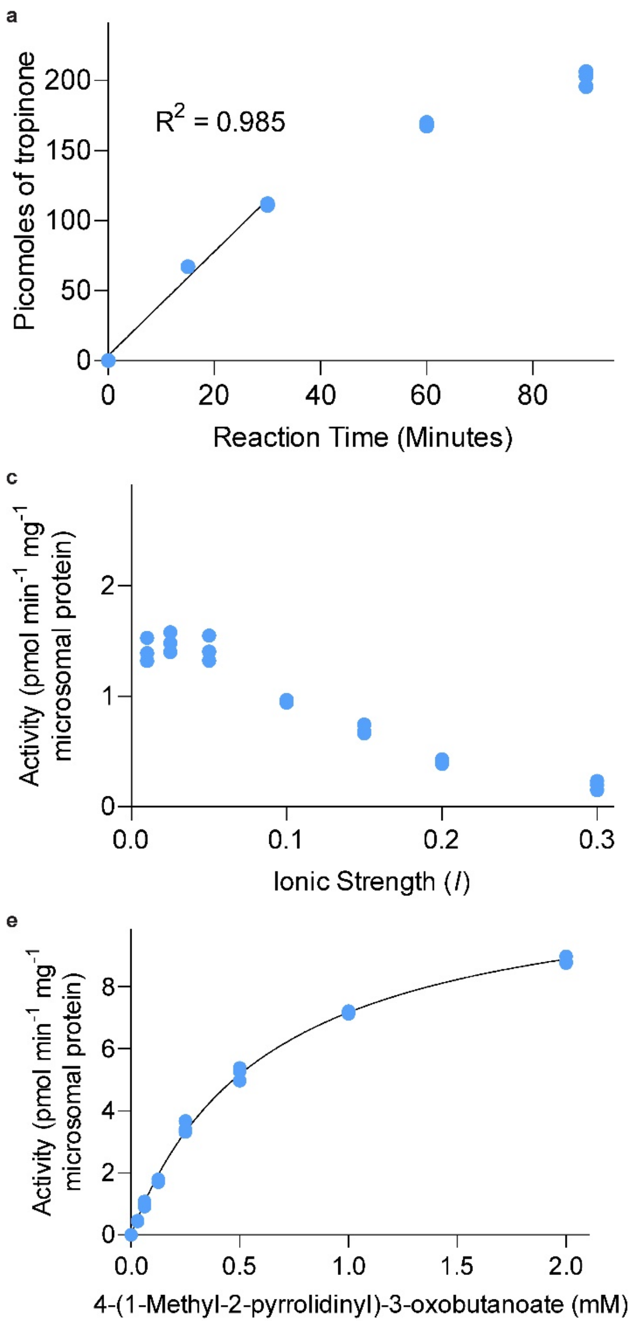

Supplementary Figure 13. Optimization and kinetic analyses of AbCYP82M3. a Determination of reaction linearity over time. **b** Determination of reaction linearity to amount of AbCYP82M3 bearing microsomal protein. **c** Determination of optimum reaction buffer ionic strength. **d** Determination of optimum pH using a buffer ionic strength of 0.05. **e** Determination of Michaelis-Menten enzyme kinetic parameters of AbCYP82M3 for 4-(1-methyl-2-pyrrolidinyl)-3-oxobutanoic acid using 1 mM NADPH as the co-substrate. Data are presented as the mean n = 3 replicates independent reactions and all individual data points are displayed as blue circles. Source data are provided as a Source Data file.

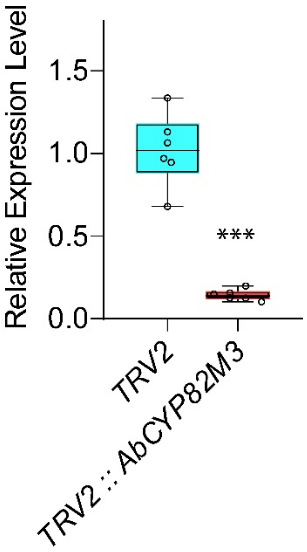

Supplementary Figure 14. Silencing efficiency in *AbCYP82M3* VIGS lines. Relative expression level of *AbCYP82M3* in *TRV2* empty vector and *AbCYP82M3* VIGS lines. Data are presented as six biological and three technical replicates with the expression level in *TRV2* empty vector control lines set to 1. The six plants selected for gene expression analysis represent individuals with tropinone levels closest to the median values for the given genotypes presented in (Fig. 5). Asterisks denote significant differences (*** = $p < 0.001$) as determined by Student's *t* test. For each box plot, the lower and upper bounds of the box indicate the first and third quartiles, the line indicates the median value, and the whiskers extend to the minimum and maximum data points. Source data are provided as a Source Data file.

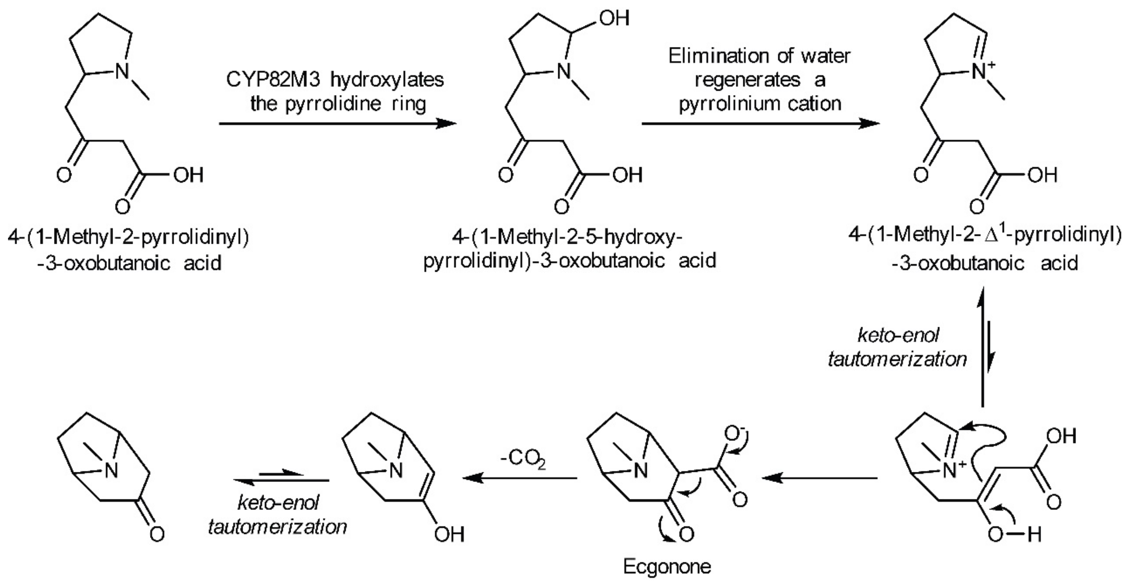

Supplementary Figure 15. Proposed mechanism for CYP82M3-mediated tropinone formation. CYP82M3 is proposed to perform a hydroxylation on the pyrrolidine ring adjacent to the nitrogen. Such a hydroxylation will lead to dehydration and the generation of a second electrophilic iminium cation. The beta-keto acid moiety of the 4-(1-methyl-2-pyrrolidinyl)-3-oxobutanoic acid compound is then capable of undergoing an intramolecular condensation reaction initiated through keto-enol tautomerization and driven to completion by decarboxylation. Further tautomerization results in formation of tropinone from its enol tautomer.

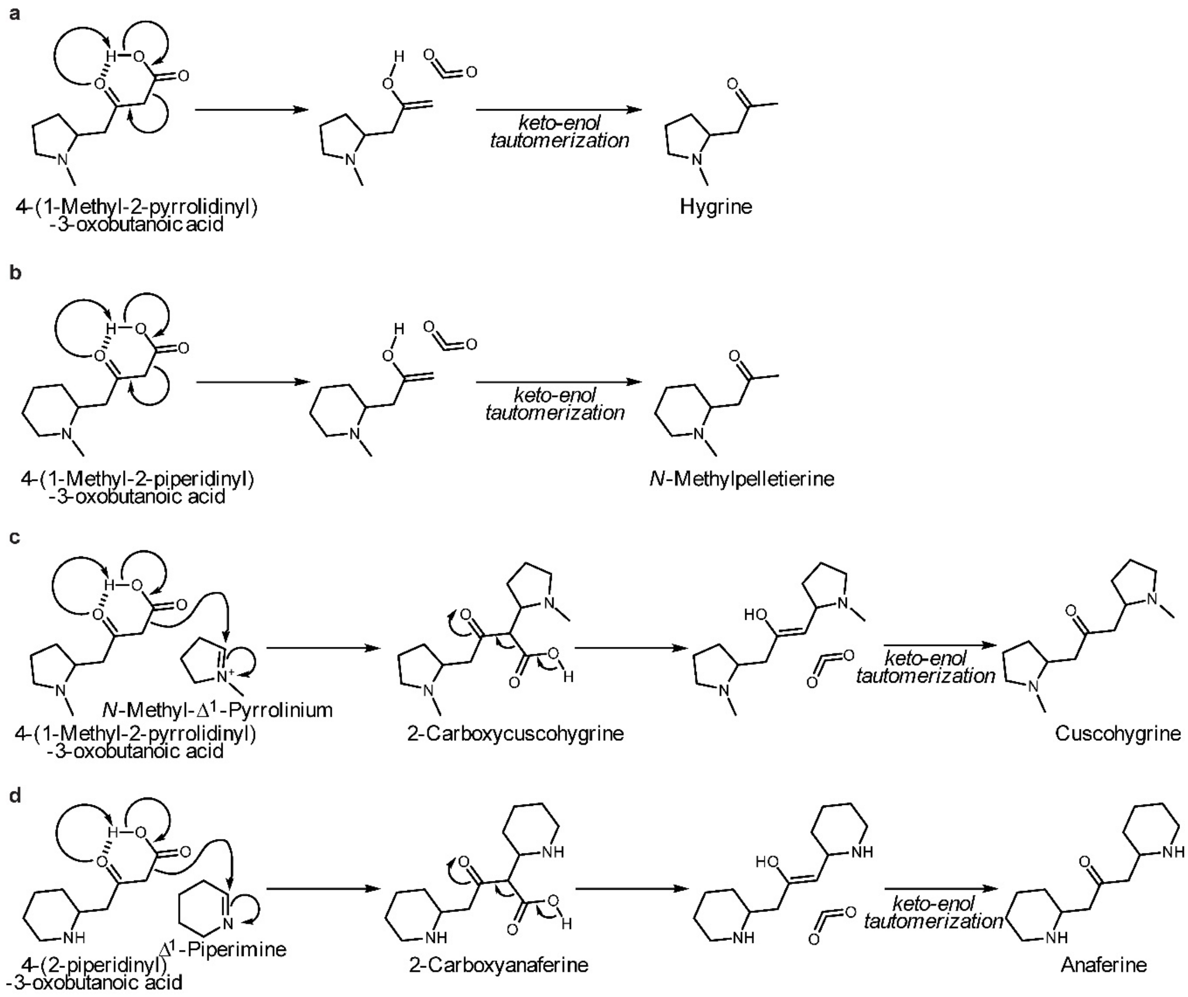

Supplementary Figure 16. Proposed mechanisms for pyrrolidine alkaloid formation. a 4-(1-Methyl-2-pyrrolidinyl)-3-oxobutanoic acid is proposed to undergo beta-keto acid decarboxylation to yield hygrine. **b** 4-(1-Methyl-2-piperidinyl)-3-oxobutanoic acid is proposed to undergo beta-keto acid decarboxylation to yield *N*-methylpelletierine. **c** 4-(1-Methyl-2-pyrrolidinyl)-3-oxobutanoic acid is proposed to yield cuscohygrine via intermolecular condensation with *N*-methylpyrrolinium through a mechanism similar to that proposed for tropinone formation. **d** 4-(1-Methyl-2-piperidinyl)-3-oxobutanoic acid is proposed to yield anaferine via intermolecular condensation with *N*-Methylpiperidinium through a mechanism analogous to that proposed for cuscohygrine formation.

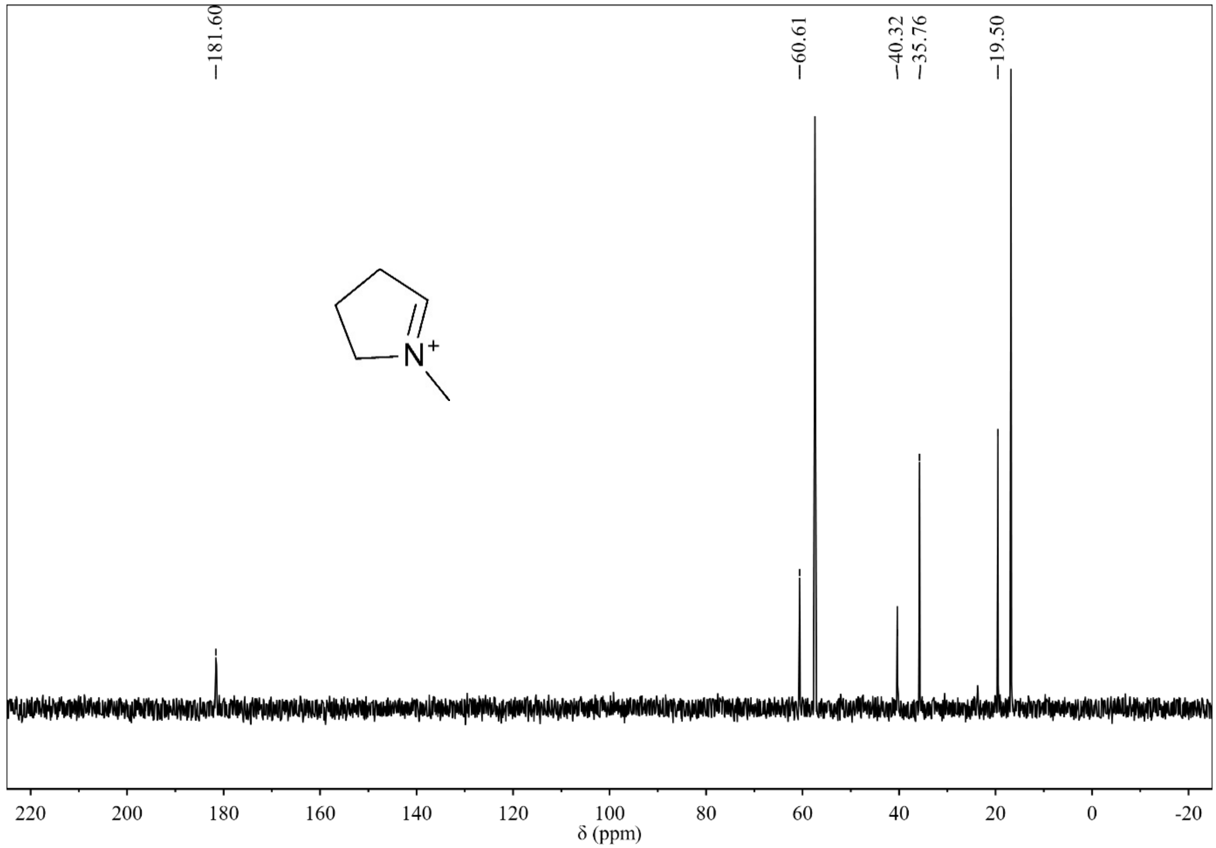

Supplementary Figure 17. ^{13}C-NMR of *N*-methyl-Δ^{1}-pyrrolinium chloride. (D_2O) δ= 19.50 (1C), 35.75 (1C), 40.32 (1C), 60.61 (1C), 181.60 (1C). NMR spectra were collected using a JEOL ECS 400 MHz NMR spectrometer.

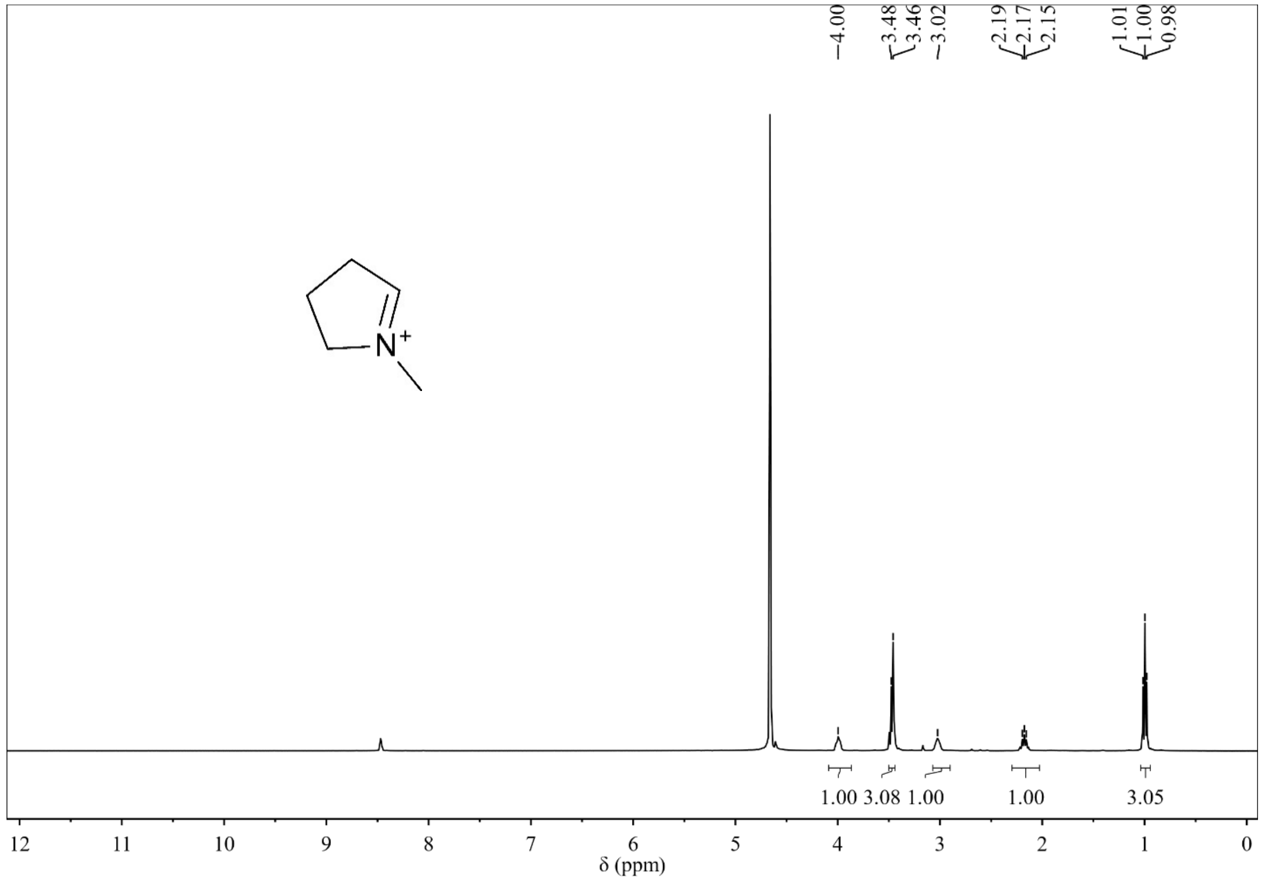

Supplementary Figure 18. ^{1}H-NMR of *N*-methyl-Δ^1-pyrrolinium chloride. (D_2O) δ= 1.00 (t, 3H), 2.17 (t, 1H), 3.02 (s, 1H), 3.46, 2.48 (d, 3H), 4.00 (s, 1H). NMR spectra were collected using a JEOL ECS 400 MHz NMR spectrometer.

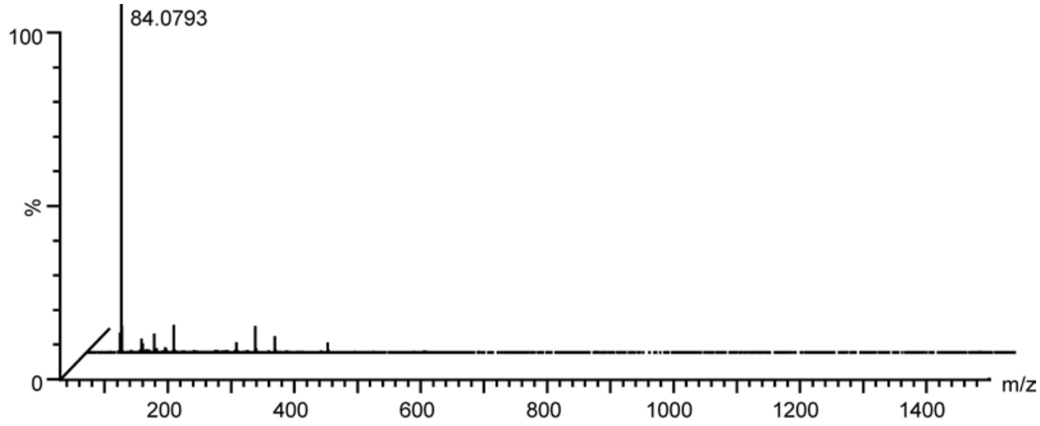

Supplementary Figure 19. High resolution mass spectrum of *N*-methyl-Δ[1]-pyrrolinium. Extracted LC-MS spectrum collected in positive-ion mode (*m/z* 84.08 ± 0.05) was generated from a 10 μM standard using a Waters Xevo G2-XS QTOF Mass Spectrometer equipped with a Shimadzu LC-20AD HPLC system and an Ascentis Express F5 column (2.1 x 100 mm with 2.7 μm particle size). Mass spectrometer conditions are the same as those used for analyses of calystegines (see methods), and chromatographic conditions are described in Supplementary Table 3.

Supplementary Tables

Supplementary Table 1. Oligonucleotide primers used in the study.

Primer Name	Sequence	Function
AbPMT2 Ent-F	5´-CACCATGGAGGTCATAAGCAACCA-3´	Expression Construct Assembly
AbPMT2 Ent-R	5´-TCAAAACTCAACCAAATCCCTCGCG-3´	Expression Construct Assembly
AbMPO2 Ent-F	5´-CACCATGGCCGCAACCTCACATAAGCC-3´	Expression Construct Assembly
AbMPO2 Ent-R	5´-TCAAAGCTTGGCCATCAAACCAGT-3´	Expression Construct Assembly
AbPYKS BamH 1 F	5´-AAGGATCCATGAAGTTGGAAAATGGTCAA-3´	Expression Construct Assembly
AbPYKS Sal 1 R	5´-AAGTCGACTTAAATGGGCACACTACGAAG-3´	Expression Construct Assembly
AbPYKS Ent-F	5´-CACCATGAAGTTGGAAAATGGTCAAAAAT-3´	Expression Construct Assembly
AbPYKS Ent-R	5´-TGCACTAGACAAGTTTCCATATTC-3´	Expression Construct Assembly
AbCYP82M3 Ent-F	5´-CACCATGWBNRAYRAYWTHWVNTTYAAY-3´	Expression Construct Assembly
AbCYP82M3 Ent-R	5´-CTAAAATTCATAAAGCACAGAATTC-3´	Expression Construct Assembly
AbCYP116623 Ent-F	5´-CACCATGGAAATTCACATTCTTTCACA-3´	Expression Construct Assembly
AbCYP116623 Ent-R	5´-TTAAACCTCATAATACACAGGAATC-3´	Expression Construct Assembly
AbCYP5021 Ent-F	5´-CACCATGGAGCTTCCATTCTCCCA-3´	Expression Construct Assembly
AbCYP5021 Ent-R	5´-TCAGGATTTATACATATAAGGAATTCCAACTAC-3´	Expression Construct Assembly
AbCYP117747 Ent-F	5´-CACCATGGAGACTATGTACCAAATAAAC-3´	Expression Construct Assembly
AbCYP117747 Ent-R	5´-CTACAGCCTGTTCAAAATTAAGTTAACACC-3´	Expression Construct Assembly
AbPYKS qRT F	5´-TGCTTCGTAGTGTGCCCATTT-3´	RT-qPCR
AbPYKS qRT R	5´-TTCTGTCTTTTCTTTGGTGGTCC-3´	RT-qPCR
AbCYP82M3 qRT F	5´-AGTTATCAAGGCAACTGTATTGAGC-3´	RT-qPCR
AbCYP82M3 qRT R	5´-ATGAGCCATGTTAGGTGAACTGC-3´	RT-qPCR
AbP450-116623 qRT F	5´-TGCAAGGGAATATTAAGGTGATGA-3´	RT-qPCR
AbP450-116623 qRT R	5´-TCTAGCCAACGCTGAAGAATAACA-3´	RT-qPCR
AbP450-5021 qRT F	5´-TGCGCCCGTGCCTCTAT-3´	RT-qPCR
AbP450-5021 qRT R	5´-TCTCATATCCACCAAGTTCACATCTT-3´	RT-qPCR
AbP450-117747 qRT F	5´-TGGACAATGTTTCTATTGAGCATACA-3´	RT-qPCR
AbP450-117747 qRT R	5´-AAGCTCGCAAAACCTCTTCTCTAG-3´	RT-qPCR
AbEF1-F	5´-CCCAAGAGGCCATCAGACA-3´	RT-qPCR
AbEF1-R	5´-ACCAATCTTGTAAACGTCCTGAAGT-3´	RT-qPCR
AbPYKS VIGS F	5´-CGACGACAAGACCCTTGCTTCGATTGGCCAAGGACTTAGC-3´	VIGS Construct Assembly
AbPYKS VIGS R	5´-GAGGAGAAGAGCCCTTGCACTAGACAAGTTTCCATATTC-3´	VIGS Construct Assembly
AbCYP82M3 VIGS F	5´-CGACGACAAGACCCTCAAGTGGCGCATCTCACAATTAGTC-3´	VIGS Construct Assembly
AbCYP82M3 VIGS R	5´-GAGGAGAAGAGCCCTTAATACTAAAGTTTGAAAAGAGTGC-3´	VIGS Construct Assembly
AbMPO2 int 1	5´-GCTGACTGTGAATCCGTGGTTAAA-3´	Sequencing Primer
AbMPO2 int 2	5´-CCGGAGGAGTAGAAACCATCGAAAA-3´	Sequencing Primer
AbMPO2 int 3	5´-GCTTGCAATTCAGACCTAAGCAGTG-3´	Sequencing Primer
AbCYP82M3 int 1	5´-ACATGTCCGAATTTCCGAGGTGGA-3´	Sequencing Primer
AbCYP82M3 int 2	5´-CTTTCTGTACCAACTTTGTTGTCTAT-3´	Sequencing Primer
AbCYP5021 int 1	5´-CTACCATCAATCTCAGCCAGATGA-3´	Sequencing Primer
AbCYP116623 int 1	5´-GCATGTTCGAATTGCAGAAGTGGA-3´	Sequencing Primer
AbCYP116623 int 2	5´-CGATCTCTACCAACTTTTGTGTCAA-3´	Sequencing Primer
AbCYP117747 int 1	5´-CAAAAGCCTCATAATCCTCTAGCCA-3´	Sequencing Primer
AbCYP117747 int 2	5´-CCAGCAGGTAGACTCATATCTCCTA-3´	Sequencing Primer

Supplementary Table 2. Multiple reaction monitoring parameters utilized for LC/MS/MS analyses of tropane and pyrrolidine alkaloids.

Compound	Precursor ion > product ion (*m/z*)	Cone voltage (V)	Collision voltage (V)	Retention time (min)
Function 1				0.0 – 1.4
Putrescine	89 > 72	22	10	0.8
N-Methylputrescine	103 > 72	22	10	0.87
Function 2				1 – 3
N-methyl-Δ^1-pyrrolinium	84 > 42	34	16	1.41
Tropinone	140.1 > 98	40	22	1.48
Hygrine	142.1 > 84	28	16	1.78
4-(1-Methyl-2-pyrrolidinyl)-3-oxobutanoic acid	186.1 > 84	28	16	1.47
Methyl (*S*)-4-(1-methylpyrrolidin-2-yl)-3-oxobutanoic acid	200.1 > 84	28	16	2.07
Function 3				1.6 – 6
Littorine	290.17 > 93	28	34	3.38
Hyoscyamine	290.17 > 93	28	34	3.15
Scopolamine	304.15 > 103	34	40	2.41
Telmisartan[a]	515.3 > 276.1	50	50	5.31

Data were analyzed in positive-ion mode using a Waters Acquity TQD Mass Spectrometer.
[a]1 µM Telmisartan was included as an internal standard.

Supplementary Table 3. UPLC mobile phase gradients utilized for LC/MS/MS analyses of tropane and pyrrolidine alkaloids using a Waters Acquity TQD mass spectrometer.

Time (min)	Mobile phase A (%)	Mobile phase B (%)
0	99	1
0.50	62.5	37.5
2	50	50
4	0	100
5	0	100
5.01	99	1
6	99	1

Mobile phase A = 100 mM ammonium formate + 1% formic acid in water. Mobile phase B = 100 mM ammonium formate + 1% formic acid in 80% methanol : 20% water. Column: Ascentis Express F5 column (2.1 x 100 mm with 2.7 μm particle size).

Supplementary Table 4. Metabolite masses (m/z values) and chromatographic retention times from LC/MS analyses of tropane and pyrrolidine alkaloids.

Compound	(*m*/*z*)	Retention time (min)
Tropine	142.12	7.4
Pseudotropine	142.12	8.3
Calystegine A3	160.09	6.6
Calystegine A5	160.09	7.5
Calystegine B1	176.09	7.58
Calystegine B2	176.09	7.66
Telmisartan[a]	515.24	2.1

Data were analyzed in positive-ion mode using a Waters Xevo G2-XS QTOF Mass Spectrometer.

[a]0.05 μM Telmisartan was included as an internal standard.

Supplementary Table 5. HPLC mobile phase gradient utilized for LC-TOF-MS analyses of tropanols and calystegines using a Waters Xevo G2-XS QTOF mass spectrometer.

Time (min)	Mobile phase A (%)	Mobile phase B (%)
0	5	95
2	5	95
3	15	85
10	22	78
11	60	40
12	60	40
12.01	5	95
14	5	95

Mobile phase A = 100 mM ammonium formate + 1% formic acid in water. Mobile phase B = acetonitrile. Column: Cortecs HILIC (2.1 x 100 mm with 1.6 μm particle size).

Supplementary Notes

Supplementary Note 1. Chemical synthesis of *N*-methyl-Δ^1-pyrrolinium chloride.

The chemical synthesis of *N*-methyl-Δ^1-pyrrolinium chloride was performed using a combination of previously described methods [2, 3, 4]. Briefly, 25.3 millimoles of 4-aminobutyraldehyde diethyl acetal (purchased from Fisher AC10326-0050) was combined with 37.9 millimoles of methyl chloroformate (Fisher AC12653-1000) in dichloromethane and the reaction was stirred in an ice bath for 10 minutes. The reaction was removed from the ice and allowed to proceed at room temperature overnight. After checking reaction progress via TLC, the organic and aqueous phases were separated with an additional extraction of the organic phase with dichloromethane. Combined organic phases were dried with sodium sulfate and the reaction products were purified using a silica gel column. Resuspended product was combined with cooled tetrahydrofuran (-20°C) and lithium aluminum hydride was slowly added. A Büchner funnel was used to remove insoluble lithium and aluminum salts following neutralization in 100 mL of water. After evaporating dichloromethane, a 95% yield was obtained. Cyclization was performed as previously described [3] with the addition of stirring the reaction overnight at room temperature. The aqueous layer was washed with ether to remove organic impurities. Water was evaporated under vacuum using a rotary evaporator, leaving the final compound in an oil state. ^{13}C, ^{1}H NMR spectra and high-resolution mass-spectra were collected to confirm product identity (Supplementary Figures 17 - 19).

Supplementary References

1. Bedewitz MA*, et al.* A root-expressed L-phenylalanine:4-hydroxyphenylpyruvate aminotransferase is required for tropane alkaloid biosynthesis in *Atropa belladonna*. *Plant Cell* **26**, 3745-3762 (2014).

2. Mizusaki S, Kisaki T, Tamaki E. Phytochemical studies on tobacco alkaloids. XII. Identification of γ-methylaminobutyraldehyde and its precursor role in nicotine biosynthesis. *Plant Physiol* **43**, 93-98 (1968).

3. Feth F, Wray V, Wagner KG. Determination of methylputrescine oxidase by high-performance liquid-chromatography. *Phytochemistry* **24**, 1653-1655 (1985).

4. Kumar P, Upadhyay RK, Pandey RK. Asymmetric dihydroxylation route to (*R*)-isoprenaline, (*R*)-norfluoxetine and (*R*)-fluoxetine. *Tetrahedron-Asymmetr* **15**, 3955-3959 (2004).
